# Variola, or Small-Pox

**Published:** 1838-07-18

**Authors:** N. Chapman

**Affiliations:** Professor of the Theory and Practice of Physic, in the University of Pennsylvania


### THE MEDICAL EXAMINER.

DEVOTED TO MEDICINE, SURGERY, AND THE COLLATERAL SCIENCES.

EDITED BY J. B. BIDDLE, M. D. AND M. CLYMER, M. D.

No. 15.] PHILADELPHIA, WEDNESDAY, JULY 18, 1838. [Vol. I.

### VARIOLA, OR SMALL-POX.

*By* N. Chapman, M. D., *Professor of the Theory and Practice of Physic, in the University of Pennsylvania.*

(Continued from page 219.)

As inoculation is a voluntary proceeding, it may be so regulated generally, as to time, and other circumstances, as to ensure the most favourable result.

The mild weather of spring and autumn, affords the best seasons; and an age not earlier than six months, should be preferred. Children, of debilitated or vitiated constitutions, are to be excluded,—except where a strong necessity exists; and the same restriction applies, indeed, to individuals of every period of life. The disease proves milder in early, than adult or more advanced age.

Formerly the preparation of the system, so called, was deemed of the utmost importance, and the same notion, even at present, is, by many, entertained. The old practice consisted in a great reduction in diet,—the avoidance of all heating, or stimulating causes,—in bleeding and purgation, and lastly, in a mercurial, and antimonial course of ten or fifteen days, or for double this period. There was, too, to use the somewhat exaggerated language of Jenner, "bleeding till the blood was thin,—purging till the body was wasted to a skeleton, and starving on vegetable diet, to keep it so." Excepting the omission of mercury, and commencing the other preparatory measures, subsequently to the act of inoculation, the same restricted and medicated plan continued, for the most part, to be pursued.

By some very able practitioners, however, the utility, and even propriety of this sort of preparation, is utterly denied. Being in a sound state, the system, it is maintained, will more effectually bear with, or resist, a morbid attack, than when the order of health is partially subverted, which it must be, by the disturbance it receives from the action of medicine, or any sudden change in the habits of life.

The correctness of this doctrine in its general application, as well as in relation particularly to the case before us, I think is amply confirmed. Cullen, who agrees with me as to the latter, says:—"I doubt on the whole, if inoculation derives any advantage from those pretended preparatory courses of medicine."

Let it not, however, be understood, that the doctrine goes so far, as to deny the necessity of an alteration in a course of living, of undue indulgence, or other bad habits. The constitution, under such circumstances, is already vitiated, and before it is infected by the disease, ought to be rectified.

Great importance was also attached to the procuring of matter from a mild case. But it having been ascertained that this has not the slightest influence, it came to be disregarded. The most lenient and distinct small-pox will produce the confluent and malignant, and conversely, the result being dependent on peculiarities of constitution, or the mode of treatment, or some other adventitious cause.

That a contrary opinion has been maintained, must not be concealed, and, I shall recite the strongest fact I have met with to sustain it. By Adams, then physician to the London Small-Pox Hospital, it is related, that adopting the notion of the power to mitigate the disease by successively inoculating from the mildest cases, he accordingly made the experiment, and, as he says, ultimately attained complete success. His plan was to select the virus from a peculiarly benignant variety of the disease, which he occasionally met with, having an eruption of a pearly-like appearance, attended by very slight constitutional disturbance, and thought, that after a long train of breeding, he had firmly and immutably fixed it as a milder stock from which to propagate. This statement, could it be received, would be entitled to great weight. But without impeaching its truth, it seems to involve a consideration, which goes far to its invalidation, or, at least, to cast over it a very dark shade of doubt. It will be perceived, that the experiment was made with a form of the disease, whose identity with genuine small-pox does not appear to have been adequately made out, or verified, and at all events, as we hear of no confirmation of it, we may presume that it proved fallacious.

In the act of inoculation, the virus is inserted under the cuticle, or rubbed into a small incision by a lancet. The other modes once practised, such as the application of a small piece of thread previously drawn through a pustule, or soaked in the matter, to a scratch, and confined by strips of adhesive plaster, were finally abandoned. From a notion that greater security was given by a multiplication of infected points, this course was pursued, two or three inoculations being performed on each arm. Camper, however, having demonstrated by a series of cautious experiments, that a single puncture was as effective, producing as large a crop of pustules, and affording equal protection, as any number up to seven, which was the highest he deemed necessary to try, we became content with a solitary inoculation. But though the quantity of virus used, be of no consequence as to the specific effect, care should be taken, not unnecessarily to enlarge or irritate the wound, as common inflammation may be set up, by which the design of the operation is defeated, and a troublesome sore ensue in its place.

On the third, fourth, or fifth day, the inoculated point begins to inflame, and at this time, is merely a small red speck, which progressively increases, till the close of the seventh day, when the constitutional symptoms manifest themselves. Be it again remarked, as deserving of attention, that there is a material difference in this respect, in the two forms of the disease. The system becomes affected, as we have seen, in the inoculated, on the seventh, and, on the fourteenth day, in natural small-pox. It teaches us, that where an individual is exposed to the infection, its operation may be superseded by inoculation, and a milder disease substituted in place of that which might otherwise take place.

The inoculated disease makes its incursion by the ordinary concomitants of pyrexia, with the addition of soreness and incipient tumefaction in the axilla of the affected arm. Great difference, however, exists in the degree of constitutional disorder. It is sometimes so slight, as hardly to be perceived, while on other occasions, it is considerable, and does, though rarely, present every gradation of violence. The case in its progressive evolution, being very similar to that of the natural disease, the history of it need not be recapitulated.

Concerning the treatment, it may suffice to state, that when mild, little is required to be done, and in the more vehement, or malignant forms, which I have said seldom occur, the course to be adopted is such, as has already been suggested, under a like circumstance, of the casual disease.

But here a question arises, which demands the most serious examination. It involves the no less momentous consideration, than what are the signs, or criteria, by which we are to determine in the case of inoculation, whether the system has been so affected by the process, as to have its susceptibility destroyed to future attacks. This is a point still so unsettled, that it is exceedingly difficult to arrive at any satisfactory conclusion, or definite and secure course of proceeding. It was, in the later periods of inoculation, a common conviction among practitioners, that a distinct pustule, with swelling in the axilla, and some febrile movement, afforded sufficient evidence of the constitutional affection, and under such a conviction, they acted accordingly in practice. The late professor Rush, especially, inculcated this doctrine. Not wanting indeed, are some, who have gone so far as to maintain, that an unusually severe attack, instead of doing away susceptibility, is rather to be construed as an indication of the endurance of such a portion of it as to endanger a future recurrence of the disease. But we have much reason to suppose neither opinion is true, and that in many cases, at least, even when the disease is naturally acquired, an infinitely stronger impression is necessary, to afford an adequate protection, and especially during an epidemic prevalence of the disease. The early writers, their immediate successors, and down to comparatively modern times, hold this language, and are filled with admonitions against a reliance on too slight an affection. It is reiterated by them, that even a considerable eruption, running regularly through its stages, and still more so in the anomalous forms of the disease, whether natural, or artificial, does not in all instances, furnish the requisite security. That a contrary opinion was held by some, cannot be denied, and more especially by those, who in the enthusiasm of the moment, were desirous to enhance the value of inoculation.

Devoted to the cause, and blinded by the spirit of controversy, they overlooked all difficulties and objections, and presented the case in the most favourable aspect, contending, that the slightest affection was adequate to the end, so completely removing all susceptibility, as to preclude for ever the danger of any repetition of attacks.

We now know this to be the illusive language of mere *votaries*, and that a careful practitioner will not fail, in suspicious cases, to renew the operation at some short interval, as a test and a safeguard.

(To be continued.)

